# Mapping five decades of high-intensity interval training research (1977–2026): database sensitivity, intellectual structure, and thematic development

**DOI:** 10.3389/frma.2026.1872122

**Published:** 2026-10-22

**Authors:** Nuri Muhammet Çelik, Ömer Aksoy, Mehmet Soyal, Ünal Can Gökmen, Burak Taş, Meltem Özağir, Erhayat Ozgür Bayazitli

**Affiliations:** 1 Department of Coaching Education, Faculty of Sports Sciences, Batman University, Batman, Türkiye; 2 Department of Coaching Education, Faculty of Sports Sciences, Istanbul Gelisim University, Istanbul, Türkiye; 3 Department of Recreation, Faculty of Sports Sciences, Istanbul Gelisim University, Istanbul, Türkiye; 4 Department of Sport Management, Faculty of Sports Sciences, Istanbul Gelisim University, Istanbul, Türkiye

**Keywords:** bibliometrics, co-citation, database sensitivity, high-intensity interval training, reproducibility, thematic evolution

## Abstract

**Objective:**

To characterize HIIT research while testing how database membership, document eligibility, and keyword metadata affect bibliometric representations of the field.

**Methods:**

Archived Web of Science (WoS), Scopus, and PubMed exports were reconstructed through documented deduplication and retrospective computational metadata screening. The primary corpus comprised 7,141 research articles published from 1977 to the March 2026 snapshot. Analyses included database sensitivity, Bradford zones and cumulative-model fit, identifier-based Lotka estimation, source-specific citations, WoS reference co-citation, author-keyword mapping, volume-balanced thematic periods, and Scopus affiliation-country collaboration.

**Results:**

Of 18,578 record instances, 8,365 unique records remained after deduplication; 1,224 were excluded under the documented rules. Annual output increased from 80 articles in 2013 to 898 in 2025. Scopus membership covered 94.17% of the primary union, compared with 71.63% for WoS and 71.92% for PubMed. Top-20 keyword sets were similar across single-source subsets (Jaccard = 0.905–1.000), whereas keyword-field and network-size choices produced 3–8 themes. The 19 core journal identities contributed 33.61% of articles. Among 24,074 Scopus author identifiers, the estimated Lotka exponent was β = 2.447; both the fitted and classical full-range models failed bootstrap goodness-of-fit tests (*p* = 0.001). WoS co-citation identified four strongly interconnected reference communities, and the primary author-keyword map contained seven themes. Including reviews increased the corpus to 8,074 and the PubMed-only contribution from 166 to 876 records.

**Conclusion:**

Broad topic visibility was comparatively stable, but database-specific contributions and thematic granularity depended on eligibility and metadata decisions. Source-resolved indicators and explicit sensitivity analyses provide a more defensible representation than an unqualified merged-data map. Findings describe bibliographic structure, not intervention effectiveness; historical search-date precision and independent screening agreement remain limitations.

## 1 Introduction

High-Intensity Interval Training (HIIT) encompasses repeated high-intensity exercise bouts separated by recovery, with considerable variation in interval duration, intensity, mode, and accumulated workload. This flexibility connects research on athletic performance with exercise physiology and cardiometabolic health, but also complicates the interpretation of a rapidly expanding literature (Buchheit and Laursen, 2013a,b; Gibala et al., 2012). Historical and physiological reviews describe the development of this research tradition without implying that all interval protocols have equivalent effects or suitability across populations (Atakan et al., 2021; Gibala and MacInnis, 2022).

Bibliometric analysis complements substantive reviews by examining publication output and relationships among documents, references, concepts, and affiliations. Performance indicators, conceptual maps, intellectual-structure analyses, and collaboration networks answer different questions and should not be treated as interchangeable evidence (Aria and Cuccurullo, 2017; Donthu et al., 2021; Zupic and Cater, 2015). In particular, a country collaboration network describes the social organization of publication activity; it does not, by itself, establish the intellectual foundations of a field.

Previous HIIT bibliometric studies already provide considerable substantive and methodological coverage. You et al. (2021) mapped health-promotion research, Liu et al. (2022) examined cardiac rehabilitation, Sun (2024) studied children and adolescents, and Li and Fang (2024) focused on women's health. More specialized analyses address oxidative stress and sleep (Coruh, 2025; Meena et al., 2025). Paucar-Uribe et al. (2026) integrated WoS and PubMed for overweight populations; therefore, multi-database integration is not intrinsically novel in this literature. Further analyses concern chronic diseases and adolescent research (Aksoy, 2026; Pramesti et al., 2026). Supplementary Table S1 compares the populations, corpus sizes, databases, methods, and conclusions of these nine directly relevant studies. This is a structured contextual comparison, not a separately registered systematic review of bibliometric studies.

The remaining opportunity is not simply to assemble more records. Database-specific coverage, search-field definitions, document-type labels, and keyword practices can change the apparent size and structure of an interdisciplinary literature (Gusenbauer and Haddaway, 2020; Mongeon and Paul-Hus, 2016). Moreover, a record absent from one query result may still be indexed on that platform. Retrieval overlap must therefore be distinguished from intrinsic indexing coverage. Similarly, concentration curves cannot demonstrate universal Bradford or Lotka behavior without an explicit model assessment, and cross-database citation counts cannot be pooled as though their provenance were irrelevant.

The present study combines a broad HIIT map with an empirical examination of these representation choices. Its contribution is fourfold: reconstruction of a traceable multi-source corpus; quantification of database membership and document-type sensitivity; separation of reference-based intellectual structure from keyword-based conceptual structure; and model-based evaluation of productivity concentration without assuming conformity. The central question is whether the broad topic profile and the finer thematic structure remain stable when the database membership, eligibility definition, and keyword input change. Publications appearing after the archived March 2026 snapshot are used only to contextualize the revision, not to update its analytical corpus.

## 2 Methods

### 2.1 Research model and search provenance

This was a bibliometric reanalysis of archived WoS Core Collection, Scopus, and PubMed exports. The bibliographic record, not a participant or intervention outcome, was the unit of analysis. PRISMA 2020 and PRISMA-S informed the reporting of identification, deduplication, and eligibility decisions; clinical risk of bias and certainty of intervention evidence were not assessed (Page et al., 2021; Rethlefsen et al., 2021). The reconstructed flow is reported transparently as retrospective computational metadata screening, rather than as a prospectively registered clinical systematic review.

The archive contained 5,316 WoS, 6,964 Scopus, and 6,298 PubMed record instances. The earlier manuscript documented searches on 24 March 2026, whereas the Scopus export filename and an archived processing log were dated 30 March 2026. These materials establish a March 2026 snapshot but do not independently resolve the precise search-to-export sequence. No new live database search was used in this reanalysis. Consequently, 2026 counts are partial and historical search-day precision is a stated provenance limitation.

### 2.2 PIO-based operationalization of the search strategy

The documented intervention block comprised seven OR-linked expressions: “high-intensity interval training,” “high intensity interval training,” HIIT, “high-intensity interval exercise,” “high intensity interval exercise,” “high-intensity intermittent exercise,” and “high intensity intermittent exercise.” Population and outcome concepts were unrestricted scope descriptors, not compulsory search blocks. Thus, PIO operationalization meant searching I, not P AND I AND O. The recorded fields were WoS Topic (TS), Scopus TITLE-ABS-KEY, and PubMed Title/Abstract. Article and English restrictions were documented for WoS and Scopus; the PubMed query documented an English restriction without an equivalent article-type filter. Full equations are retained in Supplementary Methods S1.

The fields are not equivalent. WoS Topic and Scopus TITLE-ABS-KEY include indexing components that do not map identically to PubMed Title/Abstract; the latter also includes author keywords under PubMed's field definition (National Library of Medicine, 2026). MeSH expansion and truncation were not used in the recorded queries. A common literal term block facilitates inspection of the searches but does not establish optimal recall or field equivalence. Omission of standalone HIT and broader term variants may miss relevant records. Query-recall sensitivity could not be reconstructed from exports alone and is distinct from the database-membership sensitivity tested here.

## 3 Bibliographic corpus and eligibility

Original source identifiers and metadata were preserved before merging. Records were linked first by identical within-source identifiers and then by normalized DOI. PMID and exact normalized-title links additionally required corroborating journal information, publication years within one 1 year, compatible first-author surnames, and title similarity of at least 0.90. Title-only links did not merge conflicting nonempty DOIs. PMID links with independently corroborating metadata could reconcile DOI variants. Conflicting identifiers or insufficiently corroborated matches were retained separately and logged. Year selection used the modal recorded year, with WoS, Scopus, and PubMed as the tie-breaking source order. Every retained unit is linked back to its contributing raw rows in the crosswalk.

The revised primary eligibility definition was English-language research articles relevant to HIIT or equivalent interval-exercise terminology, with a title, author information, publication year, and source. Reviews and evidence syntheses were excluded whenever a contributing source assigned such a type. This explicitly narrows the formerly ambiguous “journal article” category and limits the effect of PubMed's broader article labeling. A review-inclusive sensitivity corpus was therefore constructed rather than silently treating this choice as inconsequential. Editorials, letters, corrections, books, conference items, other nonarticle types, and source-flagged withdrawn items were excluded. Ten additional conference abstracts were identified from journal-supplement metadata.

Topical screening used inspectable lexical rules applied to titles, abstracts, author and indexed keywords, and source names. Accepted evidence comprised explicit high-intensity interval/intermittent training or exercise expressions, sprint-interval expressions in already retrieved records, or HIIT/HIIE with corroborating exercise context. Known nonexercise expansions were rejected. This did not constitute an independent comprehensive search for sprint interval training. An ordered decision hierarchy assigned one primary exclusion reason per record, avoiding double counting. The 1967 botanical record that had defined the earlier time span failed topical eligibility; the earliest retained exercise record was published in 1977.

The archive did not contain paired independent screening decisions from which reviewer agreement could be verified. Accordingly, the previous claim of two independent human screeners was not carried forward, and inter-reviewer kappa could not be calculated from the available records. The current record-level decisions are computational and auditable; they do not establish full-text clinical eligibility or independent human agreement. This restriction applies to the interpretation of the final corpus and remains relevant to author verification before resubmission.

## 4 Data collection and analytical denominators

Analyses used explicit, module-specific denominators. Publication and journal analyses used the complete reconstructed union. Author productivity used Scopus author identifiers; affiliation-country analysis used Scopus address metadata; intellectual structure used WoS cited references. These source-restricted modules were not presented as complete observations for every union record. Annual counts retained zero-publication years. Compound annual growth for the descriptive 2013–25 interval was calculated as [(P2025/P2013)∧(1/12) −1] × 100. No statistical change point at 2013 was asserted.

Journal names were standardized through exact normalized titles and title variants attached to matched records. ISSNs and all title variants were retained for inspection, but unrestricted ISSN bridging was avoided because an erroneous shared identifier could merge distinct journals. Counts refer to these documented source identities, including title-history variants linked by the archived records. Ties in source productivity were resolved alphabetically. Citation counts remained source-native: WoS and Scopus values were paired only for matched records carrying both. Missing values were not replaced by zero, and no cross-source summed citation indicator, individual-author ranking, or corpus-wide h-index was reported. For duplicate export rows within the same source, the maximum nonmissing source-native citation count was retained; citation counts were never summed across sources.

## 5 Data analysis

### 5.1 Bradford and Lotka models

Bradford zones were formed from decreasing journal productivity, choosing whole-journal cumulative cut points nearest one-third and two-thirds of the corpus. Both adjacent journal-count ratios and their geometric summary were reported. A normalized Leimkuhler cumulative model, F(r) = ln(1 + br)/ln(1 + bJ), was fitted by least squares to the observed cumulative article proportions, where *r* is journal rank and J is the number of source identities (Bradford, 1934; Leimkuhler, 1967). Root-mean-square and maximum absolute cumulative residuals quantify descriptive fit. They are not inferential *p*-values and do not establish an exact universal scattering law.

Lotka analysis counted each distinct numeric Scopus author identifier once per included Scopus article. The full observed productivity distribution, not a truncated 1–20-publication table, was fitted to p(k) = k∧(–β)/ζ(β), *k* ≥ 1, by maximum likelihood. The fitted exponent and classical β = 2 were assessed separately using a discrete Kolmogorov–Smirnov (KS) distance. Parametric bootstrap calibration used 999 replicates; β was refitted within every replicate for the estimated model, while the classical exponent remained fixed. Bootstrap *p* = (1 + number of simulated distances at least as large as observed)/1,000 (Clauset et al., 2009; Lotka, 1926). These are model-based diagnostics of the observed distribution; coauthorship dependence, finite observation windows, and imperfect author identifiers limit broader probabilistic interpretation. Abbreviated-name aggregation was retained only as a sensitivity check.

### 5.2 Intellectual, conceptual, and collaborative structure

Reference co-citation represents the frequency with which two references are cited together (Small, 1973). WoS cited-reference strings were parsed for DOIs. A reference containing multiple distinct DOIs was withheld as ambiguous, and repeated references within one article counted once. The 50 most frequently cited resolvable references were linked by co-citation and cosine-normalized weights, cij/√(ci cj) (van Eck and Waltman, 2009). Weighted Louvain community detection used resolution 1 and seed 20,261,003 (Blondel et al., 2008). All positive edges entered clustering; only a sparse strongest-link display was used in Figure 1. Reviews could appear among cited foundations even though review documents were excluded from the primary publication corpus. This DOI-resolved WoS network does not represent all references or all three databases.

**Figure 1:**
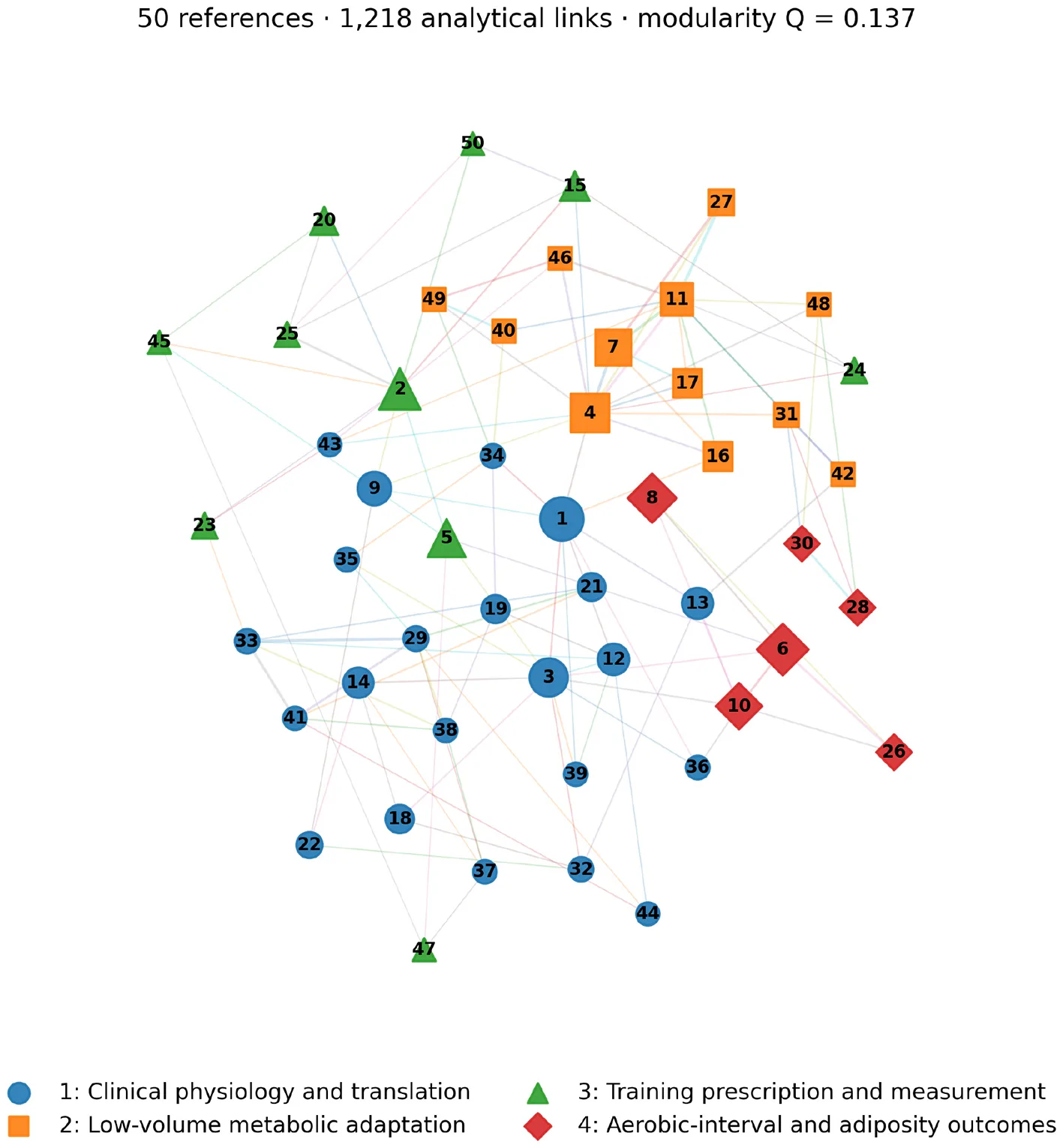
DOI-resolved intellectual structure in the WoS-member subset. Node size represents the number of included WoS articles citing a reference. Node numbers identify references in Supplementary Table S5. Communities were estimated on all 1,218 cosine-weighted edges; strongest-link sparsification is for display only. Reviews are legitimate cited foundations even when not included as primary corpus documents.

Primary co-word analysis used the union of author keywords from matched records, separated from indexed vocabulary. HTML markup, punctuation, case, diacritics, and specified lexical variants were normalized using exported dictionaries. Query-defining HIIT expressions and generic indexing or study descriptors were excluded through an explicit stoplist. Related but nonidentical expressions, such as maximal and peak oxygen uptake, were not indiscriminately merged. Document-level presence was counted once per term. The 200 most frequent informative terms occurring in at least five articles entered an association-strength network, aij = cij/(fi fj) (van Eck and Waltman, 2009), with weighted Louvain clustering at the settings above. The cap limits diagram complexity; it is a sensitivity parameter, not a claim that less frequent terms are scientifically unimportant.

Callon-style centrality and density were calculated explicitly from this weighted network: Cg = 10Σaij for edges crossing a cluster boundary, and Dg = (100/ng)Σaij for ordered within-cluster pairs (Callon et al., 1991; Cobo et al., 2011). Thus, each undirected internal edge contributes twice to density. The median cluster values defined the axes; equality was assigned to the high side. Motor, basic, niche, and emerging/declining labels describe relative network positions, not clinical maturity or demonstrated growth/decline. Exact values, member terms, and thresholds accompany the map.

Thematic periods were selected by exhaustively comparing whole-year pairs of cut points and minimizing squared deviations of keyword-bearing document counts from equal thirds. This produced 1977–2019, 2020–22, and 2023–26. The same term cap, minimum occurrence, normalization, and clustering settings were applied separately to each period. Theme links were measured by the inclusion index |A n B|/min(|A|, |B|), where A and B are cluster keyword sets (Cobo et al., 2012). All nonzero links were exported. These links measure shared vocabulary, not articles moving between periods. Alternative periods from the earlier manuscript were also examined.

Country/region labels were parsed from Scopus affiliation addresses and harmonized using a documented dictionary. Each article contributed once to every observed country; single-country publications (SCP) contained one country and multiple-country publications (MCP) at least two. Attribution was not to the corresponding author. For an article with m countries, each undirected pair received weight 2/[m(m −1)], so each multinational paper contributed one total edge unit. Country total link strength (TLS) is the sum of these fractional pair weights (Perianes-Rodriguez et al., 2016). Missing or unparsed address tails were logged.

## 6 Sensitivity analysis and reproducibility

Each single-database membership subset and each leave-one-database-out subset was compared with the primary union. Canonical pooled author keywords were held fixed for matched articles to isolate membership loss from metadata-field differences. Comparisons used article loss, top-20 keyword Jaccard similarity, full-vocabulary frequency-rank correlation, and theme count. Native database keyword availability was reported separately. Additional analyses varied the term cap (100, 200, 300), added indexed keywords, compared one-third journal cores, and included otherwise eligible reviews. These descriptive comparisons do not demonstrate query recall or equivalence of individual cluster membership.

The archived R scripts did not support a complete executable reconstruction. The revised analyses were therefore implemented in Python 3.13.5, NumPy 2.3.5, SciPy 1.17.0, and NetworkX 3.6.1, using seed 20261003 for stochastic procedures. The supplied reproduction scripts, record crosswalk, exclusion decisions, alias dictionaries, model outputs, graph edges, and cluster tables document the actual workflow. No numerical results were copied from incompatible legacy figures.

## 7 Results

### 7.1 Corpus construction and publication growth

Deduplication reduced 18,578 source instances to 8,365 unique records, removing 10,213 redundant instances. Source membership accounting identified seven within-source excess instances and 10,206 cross-source excess instances. Computational eligibility screening removed 1,224 unique records: 934 reviews/evidence syntheses, 220 nonarticle items, 44 records without confirmed exercise-related HIIT evidence, 10 supplement-derived conference abstracts, nine withdrawn items/notices, and seven records with insufficient core metadata. The primary corpus comprised 7,141 articles (Figure 2). Forty-eight records failed the topical rules overall, but four had already received an earlier exclusion reason; the flow therefore correctly assigns 44 primary topical exclusions.

**Figure 2:**
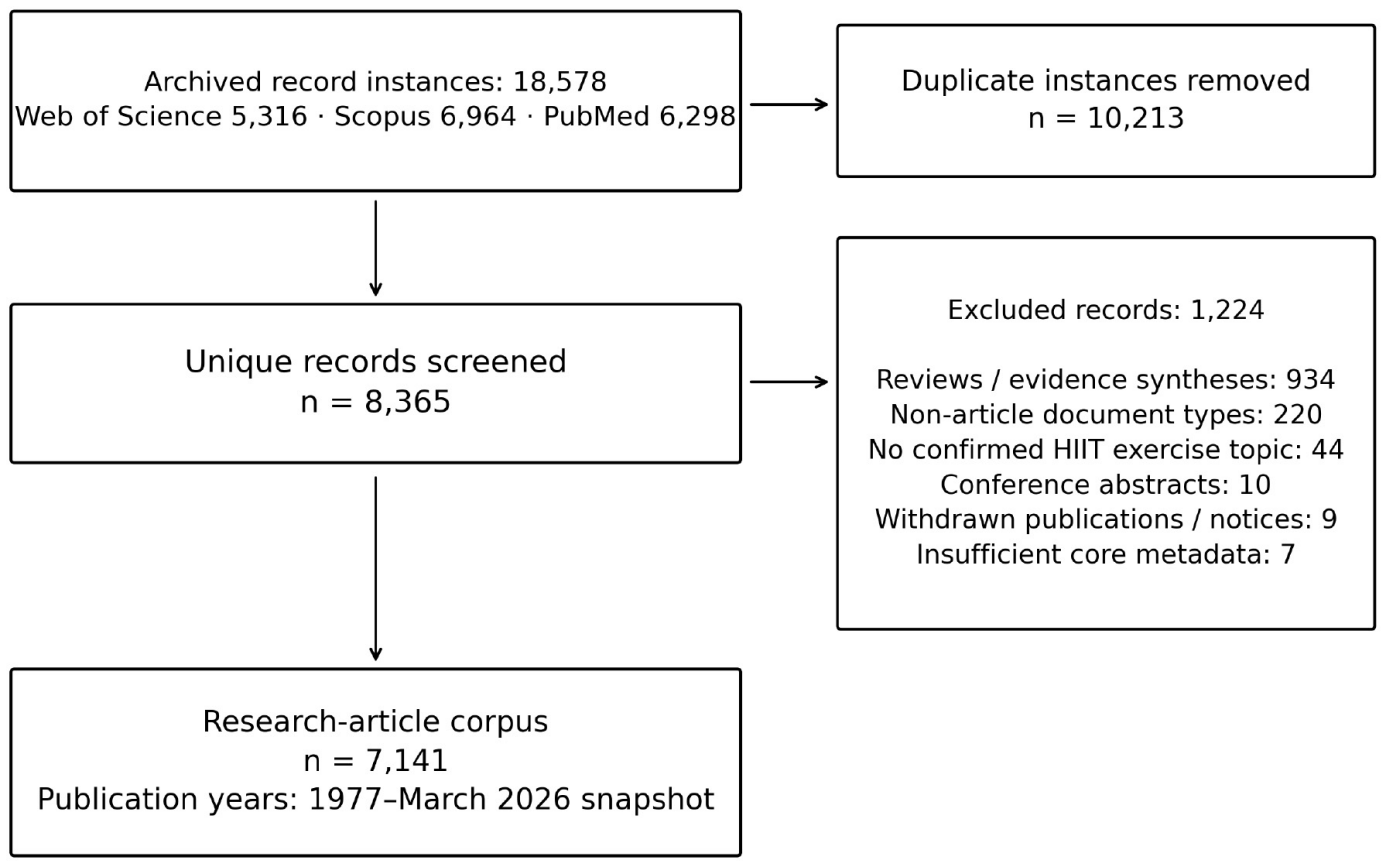
Reconstruction of the archived HIIT bibliographic corpus. The flow reports retrospective computational metadata decisions, not verified independent human screening. Each excluded unique record has one primary reason. Source instances and unique articles are distinct counting units.

The corpus extended from 1977 to the partial 2026 snapshot. Output increased from 80 articles in 2013 to 600 in 2019 and 898 in 2025, corresponding to a descriptive 2013–25 CAGR of 22.33%. The 247 records dated 2026 must not be interpreted as a complete-year decline. Supplementary Table S2 consolidates unique annual output with source-membership counts; these source columns overlap and must not be summed to obtain the union. Source-specific retrieval and metadata availability are presented in Table 1.

**Table 1:** Source-specific retrieval and metadata availability.

Source	Raw instances	Eligible articles	Union (%)	Author keywords, *n*	Cited references, *n*
WoS	5,316	5,115	71.63	4,689	5,112
Scopus	6,964	6,725	94.17	6,143	6,640
PubMed	6,298	5,136	71.92	3,865	0

Union denominator = 7,141 unique articles. Keyword and reference availability are native to each source's exported fields, before pooled metadata enrichment. Zero exported PubMed reference fields does not establish that reference information is unavailable through every PubMed-related service.

### 7.2 Database membership and eligibility sensitivity

A shared core of 4,298 articles appeared in all three saved retrievals. Exclusive pairwise memberships were WoS–Scopus (567), WoS–PubMed (79), and Scopus–PubMed (593). Database-only contributions were 171 for WoS, 1,267 for Scopus, and 166 for PubMed. Consequently, Scopus membership covered 94.17% of this research-article union, while WoS and PubMed each covered approximately 72%. These are properties of the archived queries and current eligibility rules, not platform-wide indexing recall (Table 2).

**Table 2:** Database-membership sensitivity relative to the primary union.

Subset	Articles	Loss (%)	Top-20 jaccard	Frequency ρ	Themes
WoS	5,115	28.37	0.905	0.719	7
Scopus	6,725	5.83	1.000	0.940	7
PubMed	5,136	28.08	0.905	0.720	7
Without wos	6,970	2.39	1.000	0.974	7
Without Scopus	5,874	17.74	0.905	0.802	7
Without PubMed	6,975	2.32	1.000	0.981	8

Pooled author-keyword metadata are held constant for matched records. Rank correlations include the complete union vocabulary with zero counts for absent terms. Theme counts alone are not measures of cluster equivalence.

Dominant keyword sets were similar in all three single-source subsets (Jaccard = 0.905–1.000), whereas full-vocabulary frequency correlations were lower for WoS and PubMed than for Scopus. Removing Scopus caused the largest article loss (17.74%). Eligibility had a different and pronounced effect: adding otherwise eligible reviews increased the corpus to 8,074 and the PubMed-only contribution from 166 (2.32% of the primary union) to 876 (10.85% of the expanded union). Thus, the apparent marginal contribution of PubMed changed substantially when the document-type definition changed.

### 7.3 Journal concentration, productivity models, and citations

The primary corpus contained 1,398 standardized source identities. The first Bradford zone contained 19 sources and 2,400 articles (Table 3). Frontiers in Physiology (245), European Journal of Applied Physiology (237), Journal of Strength and Conditioning Research (206), and Medicine and Science in Sports and Exercise (185) were the leading outlets. The two adjacent zone ratios differed (6.737 and 9.773), rather than reproducing an exact common multiplier. The fitted cumulative curve had a normalized RMSE of 0.0084 and maximum absolute residual of 0.0571. These values document concentration and its approximation, not exact conformity (Supplementary Figure S1).

**Table 3:** Bradford zones of the primary research-article corpus.

Zone	Source identities	Articles	Article share (%)
1	19	2,400	33.61
2	128	2,363	33.09
3	1,251	2,378	33.30

Geometric summary multiplier *n* = √(J3/J1) = 8.114; J2/J1 = 6.737; J3/J2 = 9.773. Leimkuhler parameter *b* = 0.560977. Whole-source boundaries approximate equal article thirds. Full source identities and title variants are available in the accompanying CSV.

The source-specific one-third cores contained 15 WoS, 17 Scopus, and 13 PubMed source identities. Their Jaccard similarities to the merged 19-source core were 0.789, 0.895, and 0.684, respectively. Thus, even where prominent outlets persisted, the size and composition of the operational journal core depended on source membership.

Scopus author identifiers were available for 6,722 of 6,725 Scopus-member articles. Among 24,074 distinct identifiers, 17,363 (72.12%) appeared on one article and the maximum observed productivity was 58. The fitted exponent was β = 2.447, but both the fitted and classical distributions were rejected by the model-based bootstrap KS diagnostics (Table 4; Supplementary Figure S2). Aggregation by abbreviated Scopus names produced 23,084 strings and β = 2.387, demonstrating sensitivity to the counting unit. No individual-person productivity or impact ranking was retained.

**Table 4:** Full-range discrete Lotka model diagnostics using Scopus author identifiers.

Model	β	KS distance	Bootstrap p
Estimated zeta model	2.447	0.01261	0.001
Classical inverse-square model	2.000	0.11331	0.001

*n* = 24,074 author identifiers; 999 bootstrap samples; minimum reported *p* =.001. The exponent was refitted within each bootstrap sample for the estimated model. Identifiers are not guaranteed to be perfectly disambiguated people. The bootstrap null uses idealized independent draws; coauthor dependence limits inference from these diagnostics.

For 4,865 articles with paired source-native citation counts, median WoS and Scopus counts were nine and ten. Rank correlation was high (ρ = 0.992), but Scopus counts exceeded WoS counts for 2,545 articles; WoS counts were higher for 589, and 1,731 were equal. The median paired difference was one citation. Strong rank agreement therefore did not make the two citation series numerically interchangeable.

### 7.4 Reference-based intellectual structure

Among 5,115 WoS-member articles, 5,108 contained at least one unambiguously resolved cited DOI. Of 241,197 reference strings examined, 2,241 with multiple distinct DOIs were withheld. The resolved reference universe comprised 101,309 DOIs; the 50 most frequently cited references each appeared in at least 126 articles. Their co-citation network contained 1,218 positive links and four communities of 22, 12, 10, and six references (Figure 1). Modularity was only *Q* = 0.137, indicating substantial interconnection rather than sharply separated schools.

The most frequently cited reference was (Gibala et al. 2012; 580 citing articles), followed by the cardiopulmonary programming framework of (Buchheit and Laursen 2013a; 523) and the cardiometabolic review of (Weston et al. 2014; 442). Inspection of the leading references suggested communities emphasizing clinical-physiological translation, low-volume metabolic adaptation, prescription and measurement, and aerobic-interval or adiposity outcomes. These labels are interpretive summaries of reference groupings, not independent tests of theoretical categories. The complete numbered reference key and all network edges are supplied as Supplementary Table S5 and machine-readable files.

### 7.5 Conceptual structure and thematic evolution

Author keywords were available for 6,537 articles; 6,536 retained informative terms after cleaning. The resulting vocabulary contained 10,025 terms. The 200-term primary network had 4,303 edges and seven communities (Table 5; Figure 3). Performance and oxygen-uptake terminology was connected with physical activity, fitness, molecular mechanisms, body composition, and clinical exercise contexts. Obesity and resistance training appeared within a basic theme rather than being treated as an isolated emerging topic.

**Table 5:** Verifiable thematic-map values for the primary author-keyword network.

ID/representative terms	Terms	Centrality	Density	Position
1. maximal oxygen uptake; sprint interval training; performance	52	5.850056	1.804683	Motor
2. physical activity; cardiorespiratory fitness; body composition	39	5.597673	1.341866	Motor
3. aerobic exercise; inflammation; skeletal muscle	42	5.524345	1.450758	Motor
4. obesity; resistance training; endurance training	34	5.978382	1.105598	Basic
5. blood pressure; heart rate variability; cardiac rehabilitation	17	2.564737	0.968297	Emerging/declining
6. exercise intensity; interval exercise; oxygen uptake	8	1.559427	1.504908	Niche
7. physiology; combat sports; martial arts	8	1.451610	0.903250	Emerging/declining

Median centrality = 5.524345200; median density = 1.341865736. Equality belongs to the high side: theme 2 lies on the density threshold and theme 3 on the centrality threshold. Assignments use unrounded values. “Emerging/declining” is a relative low-centrality/low-density position, not a demonstrated direction of change. All cluster members are exported.

**Figure 3:**
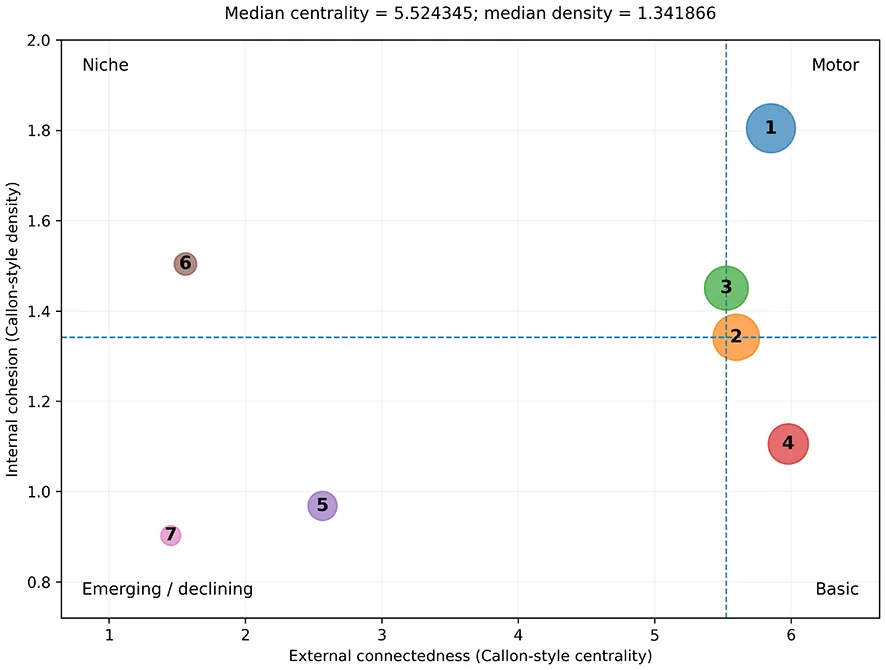
Author-keyword thematic map with explicit median thresholds. Bubble numbers correspond to Table 5; bubble size represents summed keyword document frequencies, which are not unique article counts. Axis definitions, edge counting, and the equality convention are reported in Methods. Median-boundary themes should not be interpreted as unequivocally distant from adjacent quadrants.

The number of communities changed from five to seven to eight when the author-keyword cap increased from 100 to 200 to 300. Combining author and indexed keywords at a 200-term cap produced three communities. Thus, broad topic recurrence coexisted with substantial sensitivity in thematic granularity; the seven-theme solution is not presented as a uniquely correct partition.

The selected periods contained nine, ten, and seven themes, respectively (Table 6). A first-period aging/lactate/recovery cluster shared 10 terms with the subsequent resistance-training/oxygen-uptake/fatigue cluster (inclusion index = 0.323). This is evidence of lexical redistribution, not proof that an entire clinical research program transformed into another. The earlier 1977–2018, 2019–21, and 2022–26 partition yielded 1,594, 1,785, and 3,157 informative-keyword articles and nine, nine, and eight communities. Period selection therefore materially affects the presentation. Supplementary Figure S3 summarizes selected keyword links; the complete link table permits verification without interpreting line width as publication flow.

**Table 6:** Document-volume-based periods for thematic evolution.

Period	All articles	Informative-keyword articles	Network terms	Themes
1977–2019	2,414	2,155	200	9
2020–2022	2,024	1,861	200	10
2023–2026	2,703	2,520	200	7

Whole-year cut points minimize imbalance in informative-keyword document counts. The 1977 bibliographic record remains in the corpus but lacks usable author keywords; the earliest keyword-bearing record is from 1978. The last period includes an incomplete 2026. Period-specific theme values and every positive inclusion-index link are supplied separately.

### 7.6 Affiliation-country collaboration

At least one country/region could be parsed for 6,701 of 6,725 Scopus-member articles; 2,256 (33.67%) involved multiple observed countries. There were 104 country/region labels. The United States, United Kingdom, Canada, Brazil, and Australia had the largest affiliation-based article counts (Table 7). Multinational proportions differed from output rankings; for example, Australia's proportion was 66.77% and Canada's 45.58%. The sum of country TLS values divided by two equaled 2,256, confirming the specified one-edge-unit-per-multinational-article accounting.

**Table 7:** Scopus affiliation-country publication and collaboration indicators.

Country	Articles	SCP	MCP	MCP (%)	Fractional TLS
United States	1,183	610	573	48.44	480.69
United Kingdom	733	283	450	61.39	360.74
Canada	678	369	309	45.58	251.65
Brazil	670	377	293	43.73	251.35
Australia	635	211	424	66.77	355.84
China	613	409	204	33.28	170.73
Iran	514	361	153	29.77	126.14
Spain	460	190	270	58.70	214.04
Germany	402	164	238	59.20	185.15
Norway	275	121	154	56.00	116.60

Articles are counted once per observed affiliation country, not assigned to the corresponding author. Country totals overlap. Twenty-four articles had no parsable country; 66 unparsed affiliation strings are retained for audit, including strings from otherwise partly parsable records. MCP proportions can be affected by incomplete addresses. TLS uses fractional undirected pair weights.

## 8 Discussion

### 8.1 Incremental contribution: sensitivity rather than aggregation alone

The principal contribution is a qualified answer to the representation question: dominant HIIT topic vocabulary was relatively stable under database membership changes, but database-specific contributions and fine-grained thematic organization were not invariant. The top-20 similarities should not be used to claim that all maps are equivalent; the lower full-vocabulary correlations and changing theme counts show where that inference would fail. Conversely, large differences in retrieved article numbers did not automatically imply a completely different dominant scientific vocabulary. Reporting both findings avoids treating multi-database integration as either a universal remedy or a merely procedural addition.

The review-inclusive comparison is especially informative. PubMed's unique contribution increased from 166 to 876 records when otherwise eligible reviews were included. This contrast directly illustrates the interaction between document-type harmonization and retrieval overlap. It does not show that PubMed is intrinsically deficient for research articles or intrinsically superior for synthesis. The initial search fields and article filters were asymmetric, and the present results are conditional on those saved searches. Prior analyses of academic search systems and journal coverage support the need to distinguish database characteristics from the operational search and eligibility decisions used by researchers (Gusenbauer and Haddaway, 2020; Mongeon and Paul-Hus, 2016).

Previous HIIT studies have already mapped health-oriented expansion and specific clinical or population domains (Liu et al., 2022; Paucar-Uribe et al., 2026; Sun, 2024; You et al., 2021). Their findings should not be repackaged as a new discovery simply because the present corpus is larger. The added value here is the joint demonstration that prominent vocabulary can persist while source contribution, journal-core membership, and thematic granularity change with defensible methodological choices. This distinction also explains why the database comparison is retained as a substantive methodological result throughout the abstract, aims, results, and conclusions.

### 8.2 Concentration laws and the intellectual organization of HIIT

The journal distribution shows marked concentration, but concentration is not equivalent to exact Bradford conformity. Adjacent zone multipliers were unequal, and the cumulative model's small average residual coexisted with a larger local maximum deviation. A core of 19 journals is useful for literature surveillance, yet it contains only about one-third of the analyzed articles. Restricting a systematic search to that core would therefore omit much of this reconstructed literature. The tail is substantive coverage, not statistical noise that can be discarded for convenience.

The author-identifier distribution likewise remained strongly right-skewed while rejecting both full-range Lotka models. This is a more defensible conclusion than equating a declining productivity curve with an inverse-square law. A full-range one-parameter model imposes a strong constraint on occasional and recurrent contributors simultaneously. Finite career and observation windows, collaboration, heterogeneous research specialties, and identifier errors can all contribute to departures; the current analysis does not identify their causal contributions. The difference between identifier-based and abbreviated-name exponents further shows that author disambiguation affects the distribution itself, not just a top-author list (Clauset et al., 2009; Lotka, 1926).

Reference co-citation adds an intellectual dimension that cannot be supplied by country collaboration alone. The prominence of mechanistic, programming, and cardiometabolic references is consistent with a field connected through shared physiological and methodological resources (Buchheit and Laursen, 2013a,b; Gibala et al., 2012; Weston et al., 2014). Low modularity, however, cautions against interpreting the four communities as separate paradigms. The network more plausibly depicts closely connected reference traditions. Because it is restricted to DOI-resolved WoS references, books, older non-DOI literature, and foundations disproportionately cited outside WoS may be underrepresented.

### 8.3 Conceptual interpretation and practical implications

The author-keyword map places performance, fitness, molecular mechanisms, body composition, and clinical exercise within an interconnected conceptual system. This is compatible with the breadth of HIIT applications described in physiological and historical literature (Atakan et al., 2021; Batacan et al., 2017; Gibala and MacInnis, 2022). It does not establish that a protocol is effective for every indexed condition. Moreover, generic indexed vocabulary substantially changed the partition when added to author terms. A map dominated by indexing conventions should not be interpreted as though all nodes express researchers' own conceptual priorities.

The volume-based periods reduce the extreme document imbalance of the earlier time segmentation without claiming equal calendar duration. Inclusion indices make continuity inspectable, but only at the level of shared selected vocabulary. Theme disappearance can result from a frequency threshold, a different cluster assignment, or an altered term cap rather than the abandonment of a scientific question. Accordingly, the revised interpretation avoids claims of inevitable “maturation” or directional clinical transformation unsupported by longitudinal article-level content analysis.

For research practice, the results support three concrete uses. Literature surveillance can combine core-journal monitoring with broad multi-source alerts rather than replace comprehensive searching. Bibliometric study design can specify article/review eligibility, retain source provenance, and compare metadata fields before interpreting database advantages. Collaboration planning can examine affiliation links and underlying publications to identify existing connections, while avoiding the assumption that an internationally coauthored paper necessarily has a geographically diverse participant sample. These uses are more directly supported than recommendations about the optimal intensity, duration, or clinical safety of HIIT protocols.

The citation comparison reinforces a similar boundary. Very high rank agreement did not imply equal counts, and neither measure directly evaluates study quality. Publication age, field norms, self-citation, and database visibility remain relevant. Source-separated reporting and omission of ambiguous individual-author rankings therefore improve interpretability, even though they reduce the number of headline indicators. Likewise, the country analysis describes recorded institutional participation; it is not an evaluation of national research quality or a causal explanation of unequal collaboration.

### 8.4 Limitations

First, the exact historical search-to-export chronology cannot be fully resolved: the documented search date and the export/processing dates differ within March 2026. The corpus is not a current October 2026 search and the 2026 publication year is incomplete. Second, topical and type decisions were reconstructed computationally from metadata. No independent human agreement statistic is available, and false inclusions or exclusions may remain. English-language restrictions and incomplete exports limit generalizability. The primary research-article definition is explicit but narrower than an unrestricted journal-literature map; the review-inclusive sensitivity quantifies one consequence without resolving every classification disagreement.

Third, identifier-based linkage and author counting reduce, but do not eliminate, metadata ambiguity. Conflicting identities were conservatively retained and flagged, potentially leaving residual duplicates. Source-title variants are documented rather than assumed perfectly resolved. Fourth, intellectual and country analyses use different source-specific subsets; their findings should not be extrapolated to the full union without qualification. DOI resolution omits some cited works, and partly unparsed affiliation strings may underestimate international collaboration.

Finally, term normalization, term caps, Louvain settings, median thresholds, and period boundaries shape the networks. The sensitivity analyses demonstrate this dependence but are not an exhaustive robustness study or a test of all alternative algorithms. Bootstrap Lotka diagnostics are conditional on their statistical model. No full-text intervention-quality assessment was conducted. Bibliographic association, citation prominence, and cluster position cannot establish causation, clinical effectiveness, comparative safety, or the superiority of a training prescription.

## 9 Conclusion

The reconstructed March 2026 archive supports a 7,141-article HIIT research corpus spanning 1977–2026, with marked publication growth and interconnected physiological, performance, and health-related reference and keyword structures. Its methodological contribution is the separation of relatively stable dominant vocabulary from eligibility-sensitive database contributions and metadata-sensitive thematic partitions. Bradford concentration did not imply exact geometric conformity, and identifier-based Lotka models failed full-range goodness-of-fit diagnostics. Transparent source-specific denominators, record-level decisions, fitted-model diagnostics, and accessible network tables make the revised map more verifiable while preserving the limits of the available archive.

## Data Availability

Publicly available datasets were analyzed in this study. This data can be found here: The data were retrieved from Web of Science, Scopus, and PubMed on March 24, 2026. Due to database licensing restrictions, the raw bibliographic records are not publicly available in an open repository. No accession number is applicable. The cleaned dataset and analytical workflow are available from the corresponding author upon reasonable request.
