# Supplementary material for: Mapping five decades of high-intensity interval training research (1977–2026): database sensitivity, intellectual structure, and thematic development
Source: Front Res Metr Anal. 2026 Oct 22;11:1872122. doi: 10.3389/frma.2026.1872122 (PMC13736729; doi:10.3389/frma.2026.1872122)
Supplement: Supplementary file 1 [file Data_Sheet_1.docx]

Supplementary Material

Mapping Five Decades of High-Intensity Interval Training Research (1977–2026): Database Sensitivity, Intellectual Structure, and Thematic Development

All empirical results refer to the reconstructed archived March 2026 snapshot. Contextual literature added during revision does not update that corpus.

# S1. Search documentation and provenance

The following equations reproduce the searches documented in the supplied manuscript. They are not newly executed queries. The manuscript records 24 March 2026; the Scopus export filename and processing log record 30 March 2026. The archived files do not independently settle that chronological discrepancy. Their limitations should remain visible rather than replacing either date with an unsupported common date. Search and flow reporting are documented with reference to PRISMA 2020 and PRISMA-S (Page et al., 2021; Rethlefsen et al., 2021).

## WoS Core Collection

TS=("high-intensity interval training" OR "high intensity interval training" OR HIIT OR "high-intensity interval exercise" OR "high intensity interval exercise" OR "high-intensity intermittent exercise" OR "high intensity intermittent exercise")
Recorded filters: Document type = Article; Language = English; Timespan = 1967–2026.

## Scopus

TITLE-ABS-KEY ("high-intensity interval training" OR "high intensity interval training" OR HIIT OR "high-intensity interval exercise" OR "high intensity interval exercise" OR "high-intensity intermittent exercise" OR "high intensity intermittent exercise") AND LIMIT-TO(DOCTYPE,"ar") AND LIMIT-TO(LANGUAGE,"English")

## PubMed

("high-intensity interval training"[Title/Abstract] OR "high intensity interval training"[Title/Abstract] OR HIIT[Title/Abstract] OR "high-intensity interval exercise"[Title/Abstract] OR "high intensity interval exercise"[Title/Abstract] OR "high-intensity intermittent exercise"[Title/Abstract] OR "high intensity intermittent exercise"[Title/Abstract]) AND English[Language]

PIO implementation: P and O defined unrestricted population/outcome scope; only the seven-term I block entered the recorded topical queries. Neither bibliometric-method terms nor a mandatory clinical outcome was searched. MeSH and wildcard/truncation expansion were absent. Membership sensitivity in this revision does not estimate what expanded queries would retrieve. The final empirical starting year, 1977, follows eligibility reconstruction rather than a retroactive change to the original search equation. PubMed Title/Abstract includes author keywords; an absent MeSH block is not equivalent to an absent keyword search (National Library of Medicine, 2026).

# S2. Comparison with directly relevant bibliometric studies

This structured comparison was assembled from identifiable primary publication records, targeted HIIT/bibliometric searches, and reference checks during revision. It is not claimed to be exhaustive. Corpus and method descriptions retain each study’s own scope; differences in query terms, dates, and document types prevent direct performance ranking. For Li and Fang (2024), the retrieved abstract did not specify a publication-year interval, so none is invented. Aksoy (2026) and Pramesti et al. (2026) were published after the analytical snapshot and are contextual comparators only.

*Table S1. Corpus, database, method, and contribution comparison*

| **Study** | **Corpus/scope** | Database | **Methods** | **Findings and distinction** |
| --- | --- | --- | --- | --- |
| You et al. (2021) | Health promotion; 572 publications; search window 2001–2020 | WoS | Co-citation, co-word, collaboration; CiteSpace/VOSviewer | Identifies health-oriented, mechanistic and rehabilitation directions; already maps broad health promotion. |
| Liu et al. (2022) | Cardiac rehabilitation; 201 publications; search 2000–2022; observed output 2004–2022 | WoS | CiteSpace; collaboration, keywords, citation bursts | Emphasizes cardiopulmonary fitness, coronary disease and heart failure; a clinical subdomain rather than the complete HIIT retrieval. |
| Sun (2024) | Children/adolescents; 399 publications; 2002–2022 | WoS | Co-citation, co-word and bursts; several mapping tools | Examines cardiometabolic and cognitive/mental-health directions; intellectual mapping is not new to HIIT bibliometrics. |
| Li & Fang (2024) | Women’s health; 808 publications | WoS | CiteSpace/VOSviewer; collaboration, keywords and citation patterns | Focuses on women’s health and physiological/metabolic adaptation; not a three-source sensitivity analysis. |
| Meena et al. (2025) | Oxidative stress; 192 publications; 2003–2024 | Scopus | Citation/co-citation and keyword mapping | Characterizes the oxidative-stress subdomain and its research trends. |
| Coruh (2025) | HIIT and sleep; 68 articles; 2000–2025 | WoS | Production, citation, keyword and collaboration indicators | Highlights exercise physiology, recovery and sleep-related research priorities. |
| Paucar-Uribe et al. (2026) | Overweight populations; 282 publications; 2011–2024 | WoS + PubMed | VOSviewer; keywords, collaboration and source co-citation | Already combines two databases; focuses on obesity/weight and fitness. |
| Aksoy (2026) | Chronic diseases; 1,312 English articles; 1989–2025 | WoS | VOSviewer; output, citation, keyword and collaboration maps | Describes cardiometabolic/chronic-disease themes; published April 2026, contextual only here. |
| Pramesti et al. (2026) | Adolescents; 769 publications; 2003–2024 | Scopus | Bibliometrix/RStudio and VOSviewer | Connects health/performance trends with pedagogical gaps; published September 2026, contextual only here. |
| Present reconstruction | Research articles; 7,141; 1977–March 2026 | WoS + Scopus + PubMed | Source-specific modules, fitted laws, co-citation, keyword/period maps, membership and eligibility sensitivity | Quantifies which results depend on source membership, document type and keyword input; does not claim the first HIIT map or first multi-source study. |

# S3. Reconstructed annual output and journal core

*Table S2. Annual unique output alongside overlapping database membership*

| **Year** | **Unique articles** | **WoS** | **Scopus** | **PubMed** |
| --- | --- | --- | --- | --- |
| 1977 | 1 | 0 | 1 | 0 |
| 1978 | 1 | 0 | 1 | 1 |
| 1981 | 1 | 0 | 1 | 0 |
| 1984 | 3 | 3 | 3 | 0 |
| 1988 | 1 | 1 | 1 | 0 |
| 1989 | 1 | 1 | 1 | 1 |
| 1991 | 1 | 0 | 1 | 0 |
| 1992 | 2 | 2 | 2 | 2 |
| 1993 | 3 | 2 | 3 | 2 |
| 1994 | 3 | 1 | 3 | 3 |
| 1995 | 1 | 1 | 1 | 1 |
| 1996 | 1 | 1 | 1 | 1 |
| 1997 | 8 | 6 | 8 | 1 |
| 1998 | 6 | 6 | 6 | 5 |
| 1999 | 4 | 4 | 4 | 2 |
| 2000 | 4 | 4 | 4 | 2 |
| 2001 | 3 | 3 | 3 | 3 |
| 2002 | 10 | 10 | 10 | 9 |
| 2003 | 8 | 6 | 7 | 1 |
| 2004 | 15 | 13 | 14 | 11 |
| 2005 | 10 | 9 | 9 | 10 |
| 2006 | 13 | 11 | 12 | 12 |
| 2007 | 20 | 15 | 20 | 13 |
| 2008 | 17 | 17 | 16 | 14 |
| 2009 | 31 | 29 | 31 | 28 |
| 2010 | 36 | 36 | 35 | 35 |
| 2011 | 39 | 35 | 35 | 28 |
| 2012 | 48 | 39 | 45 | 36 |
| 2013 | 80 | 70 | 76 | 67 |
| 2014 | 124 | 101 | 115 | 97 |
| 2015 | 174 | 152 | 164 | 162 |
| 2016 | 272 | 199 | 248 | 229 |
| 2017 | 380 | 269 | 355 | 299 |
| 2018 | 493 | 348 | 463 | 391 |
| 2019 | 600 | 422 | 569 | 452 |
| 2020 | 634 | 444 | 615 | 479 |
| 2021 | 701 | 473 | 675 | 474 |
| 2022 | 689 | 516 | 658 | 488 |
| 2023 | 737 | 508 | 697 | 458 |
| 2024 | 821 | 539 | 779 | 484 |
| 2025 | 898 | 663 | 848 | 645 |
| 2026 | 247 | 156 | 185 | 190 |

*Note.* Zero-publication years are retained in S01_annual_production.csv and omitted from this display only. Source columns overlap. 2026 is a partial archived year; unique annual counts total 7,141.

*Table S3. Source identities in the first Bradford zone*

| **Rank** | **Source title** | **Articles** | **Cumulative** |
| --- | --- | --- | --- |
| 1 | Frontiers in Physiology | 245 | 245 |
| 2 | European Journal of Applied Physiology | 237 | 482 |
| 3 | Journal of Strength and Conditioning Research | 206 | 688 |
| 4 | Medicine & Science in Sports & Exercise | 185 | 873 |
| 5 | International Journal of Environmental Research and Public Health | 159 | 1032 |
| 6 | PLOS ONE | 139 | 1171 |
| 7 | Physiological Reports | 131 | 1302 |
| 8 | Applied Physiology, Nutrition, and Metabolism | 130 | 1432 |
| 9 | Journal of Sports Medicine and Physical Fitness | 116 | 1548 |
| 10 | Journal of Applied Physiology | 112 | 1660 |
| 11 | Scientific Reports | 108 | 1768 |
| 12 | Nutrients | 102 | 1870 |
| 13 | International Journal of Sports Medicine | 87 | 1957 |
| 14 | International Journal of Sports Physiology and Performance | 84 | 2041 |
| 15 | Scandinavian Journal of Medicine and Science in Sports | 84 | 2125 |
| 16 | Journal of Sports Science and Medicine | 78 | 2203 |
| 17 | Journal of Physiology | 69 | 2272 |
| 18 | Journal of Sports Sciences | 67 | 2339 |
| 19 | European Journal of Sport Science | 61 | 2400 |

*Note.* Title variants, ISSNs, all 1,398 source identities, and zone assignments are exported. Journal of Strength and Conditioning Research and Strength and Conditioning Journal are separate; unrestricted shared-ISSN bridges were not used. Source counts and zones must be regenerated after any author-approved title correction.

**Figure S1. Empirical and fitted cumulative journal concentration**

Note. The Leimkuhler curve is a descriptive least-squares approximation to all ranked source identities. Its residual summaries do not constitute an inferential test of a universal Bradford law. The functional approximation follows Leimkuhler (1967).

# S4. Productivity diagnostics and keyword implementation

The KS statistic evaluates differences immediately before and after observed integer productivity values, using the theoretical discrete zeta cumulative distribution rather than a continuous KS reference distribution. The fitted-model bootstrap refits β on every simulated sample. The classical-model bootstrap keeps β = 2 fixed. Both bootstrap tests yielded .001 with 999 replicates; this is the finite-simulation reporting floor, not evidence of a zero probability. Model-based exponent intervals are not used as unconditional population confidence intervals after goodness-of-fit rejection. This separation of estimation and bootstrap goodness-of-fit evaluation follows the approach of Clauset et al. (2009).

**Figure S2. Observed Scopus author-identifier productivity and two Lotka models**

*Note.* Each numeric author identifier is counted once per article. The displayed observed range is 1–58 articles; the fitted zeta distributions have unbounded theoretical support. All frequency classes, including zeros, are available in S06_Lotka_full_identifier_distribution.csv.

Keyword preprocessing files are S08_keyword_alias_dictionary.csv and S08_keyword_stoplist.csv. Term membership for each included article is in S08_record_keywords.csv. Markup was removed before lexical normalization to avoid introducing HTML tokens into physiological terms. The primary network excludes query-defining and generic descriptors while preserving meaningful application and measurement terms. Keyword frequencies are article-level binary counts, and summed cluster frequency can exceed the number of unique articles. Weighted community detection follows Blondel et al. (2008); the thematic interpretation uses Callon-style network measures (Callon et al., 1991).

*Table S4. Keyword-field and term-cap sensitivity*

| **Field** | **Cap** | **Nodes** | **Themes** | **Median C** | **Median D** |
| --- | --- | --- | --- | --- | --- |
| Author keywords | 100 | 100 | 5 | 1.576346 | 0.579752 |
| Author keywords | 200 | 200 | 7 | 5.524345 | 1.341866 |
| Author keywords | 300 | 300 | 8 | 7.462934 | 1.879474 |
| Author + index keywords | 200 | 200 | 3 | 10.657771 | 1.958968 |

# S5. Reference key for the intellectual-structure figure

*Table S5. Numbered references in Figure 2*

| **ID** | **Reference label** | **Citing articles** | **Group** | **DOI** |
| --- | --- | --- | --- | --- |
| 1 | Gibala MJ, 2012 | 580 | 1 | 10.1113/jphysiol.2011.224725 |
| 2 | Buchheit M, 2013 | 523 | 3 | 10.1007/s40279-013-0029-x |
| 3 | Weston KS, 2014 | 442 | 1 | 10.1136/bjsports-2013-092576 |
| 4 | Burgomaster KA, 2008 | 437 | 2 | 10.1113/jphysiol.2007.142109 |
| 5 | BORG GAV, 1982 | 436 | 3 | 10.1249/00005768-198205000-00012 |
| 6 | Wisloff U, 2007 | 397 | 4 | 10.1161/circulationaha.106.675041 |
| 7 | Gibala MJ, 2006 | 384 | 2 | 10.1113/jphysiol.2006.112094 |
| 8 | Helgerud J, 2007 | 355 | 4 | 10.1249/mss.0b013e3180304570 |
| 9 | MacInnis MJ, 2017 | 331 | 1 | 10.1113/jp273196 |
| 10 | Tjonna AE, 2008 | 311 | 4 | 10.1161/circulationaha.108.772822 |
| 11 | Burgomaster KA, 2005 | 301 | 2 | 10.1152/japplphysiol.01095.2004 |
| 12 | Garber CE, 2011 | 285 | 1 | 10.1249/mss.0b013e318213fefb |
| 13 | Little JP, 2011 | 279 | 1 | 10.1152/japplphysiol.00921.2011 |
| 14 | Batacan RB Jr, 2017 | 262 | 1 | 10.1136/bjsports-2015-095841 |
| 15 | Laursen PB, 2002 | 249 | 3 | 10.2165/00007256-200232010-00003 |
| 16 | Little JP, 2010 | 231 | 2 | 10.1113/jphysiol.2009.181743 |
| 17 | Gibala MJ, 2008 | 225 | 2 | 10.1097/jes.0b013e318168ec1f |
| 18 | Ramos JS, 2015 | 223 | 1 | 10.1007/s40279-015-0321-z |
| 19 | Gillen JB, 2014 | 218 | 1 | 10.1139/apnm-2013-0187 |
| 20 | Hopkins WG, 2009 | 216 | 3 | 10.1249/mss.0b013e31818cb278 |
| 21 | Bartlett JD, 2011 | 213 | 1 | 10.1080/02640414.2010.545427 |
| 22 | Wewege M, 2017 | 182 | 1 | 10.1111/obr.12532 |
| 23 | Faul F, 2007 | 180 | 3 | 10.3758/bf03193146 |
| 24 | Tabata I, 1996 | 178 | 3 | 10.1097/00005768-199610000-00018 |
| 25 | Buchheit M, 2013 | 167 | 3 | 10.1007/s40279-013-0066-5 |
| 26 | Rognmo O, 2004 | 167 | 4 | 10.1097/01.hjr.0000131677.96762.0c |
| 27 | Burgomaster KA, 2006 | 167 | 2 | 10.1152/japplphysiol.01220.2005 |
| 28 | Boutcher SH, 2011 | 164 | 4 | 10.1155/2011/868305 |
| 29 | Jung ME, 2014 | 162 | 1 | 10.1371/journal.pone.0114541 |
| 30 | Trapp EG, 2008 | 160 | 4 | 10.1038/sj.ijo.0803781 |
| 31 | Whyte LJ, 2010 | 156 | 2 | 10.1016/j.metabol.2010.01.002 |
| 32 | Jelleyman C, 2015 | 156 | 1 | 10.1111/obr.12317 |
| 33 | HARDY CJ, 1989 | 154 | 1 | 10.1123/jsep.11.3.304 |
| 34 | Weston M, 2014 | 147 | 1 | 10.1007/s40279-014-0180-z |
| 35 | Milanovic Z, 2015 | 147 | 1 | 10.1007/s40279-015-0361-4 |
| 36 | Kessler HS, 2012 | 143 | 1 | 10.2165/11630910-000000000-00000 |
| 37 | Costigan SA, 2015 | 139 | 1 | 10.1136/bjsports-2014-094490 |
| 38 | Biddle SJH, 2015 | 139 | 1 | 10.1186/s12966-015-0254-9 |
| 39 | Kodama S, 2009 | 138 | 1 | 10.1001/jama.2009.681 |
| 40 | Gist NH, 2014 | 138 | 2 | 10.1007/s40279-013-0115-0 |
| 41 | KENDZIERSKI D, 1991 | 138 | 1 | 10.1123/jsep.13.1.50 |
| 42 | Babraj JA, 2009 | 136 | 2 | 10.1186/1472-6823-9-3 |
| 43 | Gillen JB, 2016 | 134 | 1 | 10.1371/journal.pone.0154075 |
| 44 | Craig CL, 2003 | 132 | 1 | 10.1249/01.mss.0000078924.61453.fb |
| 45 | Foster C, 2001 | 132 | 3 | 10.1519/00124278-200102000-00019 |
| 46 | Hazell TJ, 2010 | 130 | 2 | 10.1007/s00421-010-1474-y |
| 47 | Tanaka H, 2001 | 129 | 3 | 10.1016/s0735-1097(00)01054-8 |
| 48 | Talanian JL, 2007 | 129 | 2 | 10.1152/japplphysiol.01098.2006 |
| 49 | Sloth M, 2013 | 126 | 2 | 10.1111/sms.12092 |
| 50 | Billat LV, 2001 | 126 | 3 | 10.2165/00007256-200131010-00002 |

Note. Labels are transcribed from the archived reference strings and do not represent ranked individual people. The DOI is the identification key. Counts are distinct included WoS articles citing that reference; they are not global citations. Bibliographic reference-string ambiguities are separately logged. The 50 identifiers were matched to the regenerated node list; this is a corpus-reference key rather than a separate set of 50 full bibliographic records independently checked online.

# S6. Period-specific maps and verifiable transitions

*Table S6. Period-specific median thresholds*

| **Period** | **Centrality median** | **Density median** | **Themes** |
| --- | --- | --- | --- |
| 1977-2019 | 9.635095027 | 4.209894161 | 9 |
| 2020-2022 | 9.757365120 | 4.907437104 | 10 |
| 2023-2026 | 7.916973199 | 3.930762354 | 7 |

The same median/equality convention as in the primary map applies within each period. Each period’s theme table exports exact centrality, density, terms, and quadrant assignment. Because the term selection is period-specific, a theme’s absence from a subsequent map is not proof of scientific disappearance. Vocabulary continuity is interpreted within the thematic-evolution framework described by Cobo et al. (2012).

*Table S7. Selected largest vocabulary overlaps between adjacent periods*

| **Transition** | **From theme → to theme** | **Shared terms** | **Inclusion index** |
| --- | --- | --- | --- |
| 1977-2019 → 2020-2022 | aging / lactate / recovery → resistance training / maximal oxygen uptake / fatigue | 10 | 0.323 |
| 1977-2019 → 2020-2022 | physical activity / aerobic exercise / endurance training → physical activity / cardiorespiratory fitness / endurance training | 6 | 0.214 |
| 1977-2019 → 2020-2022 | resistance training / cardiorespiratory fitness / type 2 diabetes → physical activity / cardiorespiratory fitness / endurance training | 6 | 0.176 |
| 1977-2019 → 2020-2022 | sprint interval training / exercise intensity / interval exercise → heart rate / affect / cortisol | 5 | 0.385 |
| 1977-2019 → 2020-2022 | maximal oxygen uptake / cycling / peak oxygen uptake → resistance training / maximal oxygen uptake / fatigue | 5 | 0.250 |
| 2020-2022 → 2023-2026 | resistance training / maximal oxygen uptake / fatigue → resistance training / maximal oxygen uptake / sprint interval training | 22 | 0.629 |
| 2020-2022 → 2023-2026 | performance / heart rate variability / mitochondria → resistance training / maximal oxygen uptake / sprint interval training | 11 | 0.550 |
| 2020-2022 → 2023-2026 | cardiac rehabilitation / physical exercise / quality of life → quality of life / rehabilitation / cardiac rehabilitation | 9 | 0.375 |
| 2020-2022 → 2023-2026 | obesity / body composition / aging → inflammation / aerobic exercise / oxidative stress | 8 | 0.348 |
| 2020-2022 → 2023-2026 | type 2 diabetes / insulin resistance / diabetes mellitus → body composition / type 2 diabetes / mitochondria | 6 | 0.353 |

*Note.* Selected by number of shared terms, with inclusion index breaking ties. All positive connections, including less prominent links, are in S11_all_theme_transitions.csv. Inclusion index = shared terms divided by the smaller cluster’s term count; one theme can connect to several subsequent themes.

**Figure S3. Selected keyword-overlap links across document-volume-based periods**

*Note.* This is a thematic-transition display, not a mass-conserving Sankey diagram of articles. Line width represents shared selected keywords. At most two strongest outgoing links per theme, with at least three shared terms, are displayed; all nonzero analytical links remain available in the CSV.

# S7. Files required to verify and reproduce the analysis

record_decisions.csv contains every reconstructed unique record, source membership, DOI variants, topic evidence, and its single primary decision. raw_to_unique_crosswalk.csv links the raw export rows to these records. identifier_conflicts_kept_separate.json records conservative unresolved linkage cases. These are retrospective computational outputs, not the original independent reviewer decisions.

S01–S12 CSV files contain annual counts, source identities, Bradford fits, source-specific citations, full identifier productivity, affiliation-country assignments and edge weights, keyword dictionaries, thematic values, sensitivity results, period maps, complete transitions, and co-citation nodes and edges. Figure reference IDs follow the node CSV row order. all_results.json supplies the unrounded reported summaries. The runnable Python scripts and environment requirements accompany the package; the user’s original HIIT.zip is an input and is not replaced by these derived tables.

The primary corpus must not be confused with either the 8,547-record legacy merge or the 8,074-record review-inclusive sensitivity corpus. The executable pipeline regenerates the primary results from the raw export files. Independent author review of eligibility, historical chronology, source-title mappings, and final declarations remains necessary; the package does not certify that this review has already occurred. A fresh end-to-end rerun from checksum-matched raw exports reproduced the numerical results. The verification log distinguishes computational reproducibility from independent human eligibility assessment.

# References

Aksoy, Ö. (2026). High-intensity interval training (HIIT) and chronic diseases: An emerging trend in the scientific literature? A bibliometric analysis. *Istanbul Gelisim University Journal of Health Sciences, 28*, 314–329. https://doi.org/10.38079/igusabder.1822957

Blondel, V. D., Guillaume, J.-L., Lambiotte, R., & Lefebvre, E. (2008). Fast unfolding of communities in large networks. *Journal of Statistical Mechanics: Theory and Experiment, 2008*(10), P10008. https://doi.org/10.1088/1742-5468/2008/10/P10008

Callon, M., Courtial, J. P., & Laville, F. (1991). Co-word analysis as a tool for describing the network of interactions between basic and technological research: The case of polymer chemistry. *Scientometrics, 22*(1), 155–205. https://doi.org/10.1007/BF02019280

Clauset, A., Shalizi, C. R., & Newman, M. E. J. (2009). Power-law distributions in empirical data. *SIAM Review, 51*(4), 661–703. https://doi.org/10.1137/070710111

Cobo, M. J., López-Herrera, A. G., Herrera-Viedma, E., & Herrera, F. (2012). SciMAT: A new science mapping analysis software tool. *Journal of the American Society for Information Science and Technology, 63*(8), 1609–1630. https://doi.org/10.1002/asi.22688

Coruh, Y. (2025). Bibliometric analysis of articles on high intensity training (HIIT) and sleep in the Web of Science database. *Journal of ROL Sport Sciences, 6*(4), 911–924. https://doi.org/10.70736/jrolss.1720

Leimkuhler, F. F. (1967). The Bradford distribution. *Journal of Documentation, 23*(3), 197–207. https://doi.org/10.1108/eb026430

Li, Y., & Fang, J. (2024). The impact of high-intensity interval training on women’s health: A bibliometric and visualization analysis. *Medicine, 103*(39), e39855. https://doi.org/10.1097/MD.0000000000039855

Liu, H., Liu, F., Ji, H., Dai, Z., & Han, W. (2022). A bibliometric analysis of high-intensity interval training in cardiac rehabilitation. *International Journal of Environmental Research and Public Health, 19*(21), 13745. https://doi.org/10.3390/ijerph192113745

Meena, A., Chahar, P. S., & Singh, J. (2025). Analyzing research trends on high intensity interval training and oxidative stress: A bibliometric analysis (2003–2024). *Physical Education Theory and Methodology, 25*(2), 407–415. https://doi.org/10.17309/tmfv.2025.2.23

National Library of Medicine. (2026, September 30). *PubMed user guide*. Retrieved October 3, 2026, from https://pubmed.ncbi.nlm.nih.gov/help/

Page, M. J., McKenzie, J. E., Bossuyt, P. M., Boutron, I., Hoffmann, T. C., Mulrow, C. D., Shamseer, L., Tetzlaff, J. M., Akl, E. A., Brennan, S. E., Chou, R., Glanville, J., Grimshaw, J. M., Hróbjartsson, A., Lalu, M. M., Li, T., Loder, E. W., Mayo-Wilson, E., McDonald, S., … Moher, D. (2021). The PRISMA 2020 statement: An updated guideline for reporting systematic reviews. *BMJ, 372*, n71. https://doi.org/10.1136/bmj.n71

Paucar-Uribe, J. D., Rendón-Sanchéz, A. J., Peña-Giraldo, M. V., Forero González, K. R., Sanabria Moreno, A. T., Becerra-Patiño, B. A., Prieto Mondragon, L. D. P., Olivares-Arancibia, J., Yáñez-Sepúlveda, R., & López-Gil, J. F. (2026). Mapping scientific research on high-intensity interval training (HIIT) in overweight populations (2011–2024). *Sports, 14*(1), 38. https://doi.org/10.3390/sports14010038

Pramesti, F. A. A., Razak, P. N., Iswara, E. W. F., S, A., & Mukarromah, S. B. (2026). Global trends in high-intensity interval training research adolescents: A bibliometric analysis. *Indonesian Journal of Physical Education and Sport Science, 6*(3), 690–710. https://doi.org/10.52188/ijpess.v6i3.2176

Rethlefsen, M. L., Kirtley, S., Waffenschmidt, S., Ayala, A. P., Moher, D., Page, M. J., Koffel, J. B., & PRISMA-S Group. (2021). PRISMA-S: An extension to the PRISMA statement for reporting literature searches in systematic reviews. *Systematic Reviews, 10*, 39. https://doi.org/10.1186/s13643-020-01542-z

Sun, F. (2024). Frontiers and hotspots of high-intensity interval exercise in children and adolescents: Text mining and knowledge domain visualization. *Frontiers in Physiology, 15*, 1330578. https://doi.org/10.3389/fphys.2024.1330578

You, Y., Li, W., Liu, J., Li, X., Fu, Y., & Ma, X. (2021). Bibliometric review to explore emerging high-intensity interval training in health promotion: A new century picture. *Frontiers in Public Health, 9*, 697633. https://doi.org/10.3389/fpubh.2021.697633
